# Innovation in reconstructive plastic surgery

**DOI:** 10.1016/j.clinsp.2026.101108

**Published:** 2026-09-06

**Authors:** Fábio F. Busnardo, Nivaldo Alonso, Rolf Gemperli

**Affiliations:** Hospital das Clínicas da Faculdade de Medicina da Universidade de São Paulo (HCFMUSP), São Paulo, SP, Brazil

Innovation in healthcare may be understood as the invention, adoption, diffusion, and incorporation of new ideas, technologies, techniques, drugs, and care models that generate value for patients and the health system. It differs from pure technological development in that its ultimate purpose is not sophistication for its own sake, but the improvement of clinical outcomes, safety, and patient experience. Its goal is to rationalize care. When conducted properly, it directly affects the quality of care, reducing complications, shortening hospital stays, facilitating functional recovery, and enhancing the patient’s journey. At the same time, it has the potential to improve cost-effectiveness by directing resources toward interventions that produce more health per unit of investment.^1^^,^^2^ It must be acknowledged, however, that not every novelty is, by definition, cost-effective: technologies may increase expenditure without a proportional benefit, which is why health technology assessment has become a necessary step prior to incorporation. In this broader perspective, the *Lancet* Commission on Global Surgery itself demonstrated that expanding access to safe surgical procedures, far from being a luxury, constitutes a cost-effective investment and a driver of economic development.^3^ To innovate in healthcare is, therefore, to reconcile the pursuit of the state of the art with the responsibility of rendering it sustainable.

In this scenario, plastic surgery occupies a singular position. Contrary to the popular perception that reduces it to its aesthetic dimension, the specialty is devoted to the restoration of form and function in congenital malformations, sequelae of trauma and burns, defects following oncologic resections, and complex tissue losses. Its impact on the general population is broad: it restores the capacity to eat, speak, see, walk, and reintegrate socially, with profound repercussions on quality of life and productivity. Moreover, the specialty has historically been a cradle of innovation that exceeds its own boundaries. The most eloquent example is that of the North American plastic surgeon Joseph E. Murray. While treating severely burned soldiers during World War II, Murray observed that skin grafts were rejected unless they originated from identical twins, a seminal observation in the immunology of transplanted tissues.^4^ This clinical insight, born of wound reconstruction, led him to investigate organ transplantation and culminated, on December 1954, in the first successful kidney transplant in history, performed between twins at the *Brigham Hospital* in Boston.^5^ For this body of work, which opened the field of organ transplantation, Murray received the Nobel Prize in Physiology and Medicine in 1990. It is emblematic that one of the greatest revolutions in twentieth-century medicine sprang from the hands of a reconstructive plastic surgeon.

Future prospects prolong this innovative tradition. Xenotransplantation, the use of organs and tissues from genetically modified animals, is re-emerging as a possible response to the chronic donor shortage. Recent advances in the gene editing of pigs, incorporating multiple modifications intended to avert hyperacute rejection, have enabled the first transplants of porcine kidney and heart into humans.^6^ For reconstructive plastic surgery, the horizon is particularly promising: the skin of genetically modified pigs could serve as a biological cover in major burn patients, and its potential is under discussion for flaps and even for vascularized composite allotransplants, such as face transplants.^7^ In this context, it is no coincidence that the University of São Paulo houses a xenotransplantation research center with a line dedicated to skin grafts.^7^

Another area of potential innovation in plastic surgery is robotic surgery. The method is already well established across several surgical specialties. In Brazil, this movement has even reached the public health system, with the incorporation, within the Unified Health System (SUS), of robot-assisted radical prostatectomy for patients with prostate cancer.^8^ In plastic surgery, however, its application remains incipient, concentrated in institutional protocols, clinical series, and a few specialized centers, with evidence still being consolidated, although there is growing interest in areas such as microsurgery, supermicrosurgery, breast reconstruction, and the reconstruction of anatomical regions that are difficult to access.^9^^,^^10^ In this context, robotic surgery increases the precision of surgical gestures through improved visualization, tremor filtering, and motion scaling; it favors microsurgery and minimally invasive flap dissection, with the potential to reduce donor-site morbidity; and it extends the specialist's dexterity into narrow or deep anatomical fields. It is, therefore, a technology that, integrated into reconstructive surgery, promises to progressively redefine the limits of what is technically possible in the restoration of form and function.^11^

A potentially more far-reaching application of robotic surgery for a country of continental dimensions such as Brazil may be telesurgery. Since the historic “Lindbergh Operation” in 2001, when a cholecystectomy was conducted remotely between New York and Strasbourg,^12^ the consolidation of fiber-optic networks and 5 G technology has drastically decreased latency and rekindled the field. In 2024, a pioneering telesurgery connection linked São Paulo to Orlando in an experimental model,^13^ and, in 2025, transcontinental procedures were performed on patients thousands of kilometers away. From this evolution, consider a future to come: an Indigenous child with a cleft lip, born in a village in the Amazon, being operated on by a specialist controlling the robotic system from the Hospital das Clínicas of the University of São Paulo Medical School (HCFMUSP). This is no mere exercise of imagination. Cleft lip and palate are the most common congenital malformation, with an approximate incidence of 1.5 per thousand live births, and its surgical treatment is marked by profound regional inequalities ‒ the highest rates of delay are concentrated in the North and Northeast regions, where specialized infrastructure is scarce.^14^ Telesurgery offers a path to bring the excellence of major centers closer to historically underserved populations.

There remains, nonetheless, the greatest challenge of all in innovation: access. The frontier of knowledge is of little value if its fruits remain restricted to a few. It is estimated that five billion people worldwide lack access to safe and sustainable surgical care, a burden that falls above all on the poorest, the marginalized, and the geographically isolated.^1^ An innovation that reaches only the privileged strata not only fails in its purpose but also deepens the inequity it ought to combat. Brazil offers examples of the opposite path, in which public policy and innovation converge: Law n° 15,133 of 2025 made reconstructive surgery for cleft lip and palate by the Unified Health System mandatory nationwide. The true test of innovation in reconstructive plastic surgery will therefore not be the sophistication of xenotransplantation, the robot, or telesurgery in themselves, but our capacity to ensure that these achievements reach all strata of the population, from the major academic center to the most remote village. To innovate, in the final analysis, is also to democratize. This must be the ethical and scientific compass that guides the specialty's future.

## Data availability

Not applicable.

## Declaration of competing interest

The authors declare no conflicts of interest.
